# Restoring morphology of light sheet microscopy data based on magnetic resonance histology

**DOI:** 10.3389/fnins.2022.1011895

**Published:** 2023-01-04

**Authors:** Yuqi Tian, James J. Cook, G. Allan Johnson

**Affiliations:** Department of Radiology, Duke University School of Medicine, Durham, NC, United States

**Keywords:** mouse brain imaging, magnetic resonance histology, light sheet microscopy, cross-modality registration, tissue clearing

## Abstract

The combination of cellular-resolution whole brain light sheet microscopy (LSM) images with an annotated atlas enables quantitation of cellular features in specific brain regions. However, most existing methods register LSM data with existing canonical atlases, e.g., The Allen Brain Atlas (ABA), which have been generated from tissue that has been distorted by removal from the skull, fixation and physical handling. This limits the accuracy of the regional morphologic measurement. Here, we present a method to combine LSM data with magnetic resonance histology (MRH) of the same specimen to restore the morphology of the LSM images to the in-skull geometry. Our registration pipeline which maps 3D LSM big data (terabyte per dataset) to MRH of the same mouse brain provides registration with low displacement error in ∼10 h with limited manual input. The registration pipeline is optimized using multiple stages of transformation at multiple resolution scales. A three-step procedure including pointset initialization, automated ANTs registration with multiple optimized transformation stages, and finalized application of the transforms on high-resolution LSM data has been integrated into a simple, structured, and robust workflow. Excellent agreement has been seen between registered LSM data and reference MRH data both locally and globally. This workflow has been applied to a collection of datasets with varied combinations of MRH contrasts from diffusion tensor images and LSM with varied immunohistochemistry, providing a routine method for streamlined registration of LSM images to MRH. Lastly, the method maps a reduced set of the common coordinate framework (CCFv3) labels from the Allen Brain Atlas onto the geometrically corrected full resolution LSM data. The pipeline maintains the individual brain morphology and allows more accurate regional annotations and measurements of volumes and cell density.

## 1. Introduction

Combining mesoscopic structural information of the brain and histology at the cytoarchitectural scale has been a focus in recent years to reveal the bridge between tissue morphological alternations and disease (Casanova et al., 2009; Vemuri and Jack, 2010; Zhang et al., 2012), brain insult (Tuor et al., 2014; Fornito et al., 2015; Weishaupt et al., 2016) and aging (Eylers et al., 2016; Schmitz et al., 2018). There is clear evidence that morphological disruptions underlie brain dysfunctions at both the meso- and microscopic scale; for example the corpus callosum volume reduction in autism (Egaas et al., 1995; Hardan et al., 2000; Tepest et al., 2010; Loomba et al., 2021) and neuronal death following ischemic insult (Weishaupt et al., 2016). Merging structural changes in specific brain regions at the mesoscale with corresponding quantitative cellular measurements at the microscopic scale will open an entirely new window into understanding the brain.

Diffusion tensor imaging (DTI) provides particularly unique insight into brain morphology and connectivity (Fornito et al., 2015). However, extension of DTI to more basic studies in the mouse is challenging because the mouse brain @ 435 mg is about 3,000 times smaller than the human brain. Through a series of innovations, the Duke Center for *in vivo* Microscopy (CIVM) has extended the spatial resolution of magnetic resonance imaging (MRI)/DTI by more than 500,000 times that of routine clinical scans in perfusion fixed post mortem specimens (e.g., MRH) (Johnson et al., 1993; Johnson et al., 2007). Recent work has pushed the resolution of DTI to 15 × 15 × 15 μm^3^ and accelerated the acquisition with compressed sensing, which enables routine acquisition of high-resolution multidimensional whole mouse brain images (Wang et al., 2018a; Johnson et al., 2019, 2022). These high-fidelity mesoscale MRH data now enable correlation between the MRH metrics and the tissue cytoarchitecture.

The development of tissue clearing and LSM have allowed neuroscientists to routinely image whole cleared mouse brains at cellular resolution (Erturk et al., 2012). Continued innovation in clearing (SHIELD) (Park et al., 2019) and immunohistochemistry (SWITCH) (Murray et al., 2015) has enabled staining of varied cell types (neuron, oligodendrocyte, microglia), structural proteins (myelin) and pathologies (a-beta and tau proteins).

**FIGURE 1:**
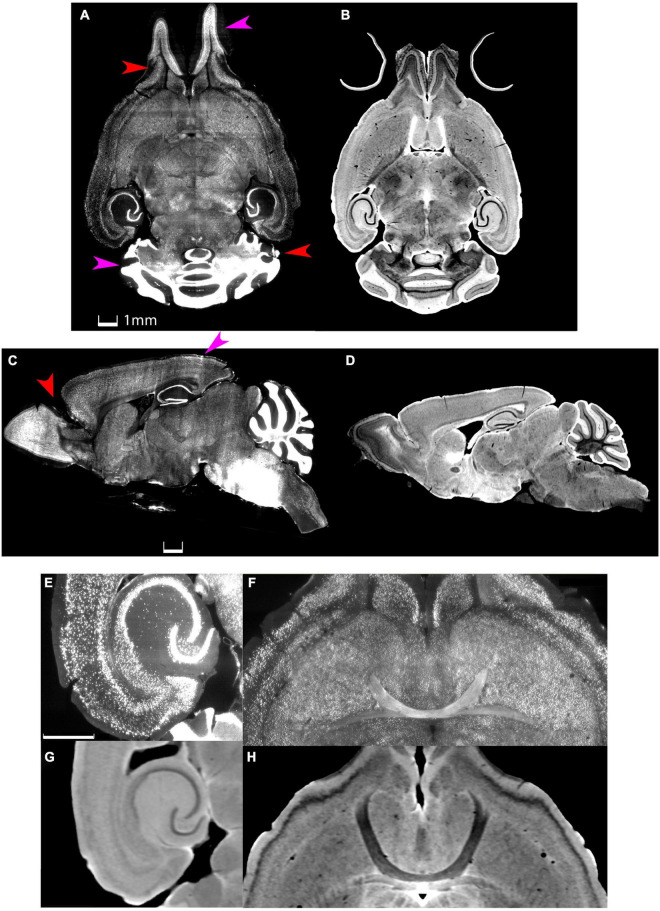
Distortion and tissue tearing in light sheet microscopy (LSM) compared to magnetic resonance histology (MRH). A comparison between LSM images of a mouse brain stained with NeuN **(A,C,E,F)** and a diffusion weighted MRH image of the same specimen **(B,D,G,H)** highlights some of the challenges and opportunities. Red arrows indicate the tissue tearing. Purple arrows indicate the swelling (specimen 200316). Scale bar: 1 mm.

Merging MRH and LSM data from the same specimen will capture the best of both. MRH with DTI is a non-destructive and multi-contrast imaging method which preserves accurate brain morphology since the scanning can be done with the brain in the skull. DTI with high angular sampling provides maps of whole brain connectivity (Johnson et al., 2019). Multiple scalar images provide exquisite tissue contrast differentiating brain subunits. Post processing pipelines can exploit these multi-contrast images to automatically label more than 300 different sub-regions (Johnson et al., 2022). LSM provides cellular resolution but requires the removal of the brain from the skull and tissue clearing, which induces tissue swelling. Dissection of the brain from the skull frequently results in tissue loss or tearing (Figure 1). Labeling is not always as uniform as one might hope. Mapping LSM to MRH restores the tissue geometry and allows automated labeling of the sub-regions in the LSM data.

Finally, the most common method for labeling cleared brain images (Kutten et al., 2016; Goubran et al., 2019; Tappan et al., 2019; Perens et al., 2021) involves registration to the Allen Brain Atlas which has been constructed from 2D serial sections acquired at 100 μm intervals averaged from ∼1,600 young adult C57BL/6J mice (Wang et al., 2020). Mapping the cleared brain images from another strain at a different age to the ABA may obscure regional volume changes that might be important image phenotypes for the study.

Our long-range goal is development of the infrastructure to support routine, comprehensive morphologic phenotyping of the mouse brain using combined MRH and LSM to map the genetic impact on cells and circuits. Those familiar to registration methods will appreciate that registration of images into a common space requires recognition of the challenges that are unique to the task and adapting the code to those challenges. Those challenges are: (1) The sources of contrast in MRH and LSM are wildly different. (2) Each modality has many different contrasts, e.g., 11 different scalar images in MRH and even greater number of contrasts in immune histochemistry for LSM. (3) The geometric distortion in the LSM data can exceed 40% and there is frequent tissue loss. (4) The data volumes are large approaching a terabyte for a single specimen. In this paper we have addressed a these challenges, developed a process for optimizing the software, and highlighted some of the limitations in combining MRH/LSM of the same brain routine.

## 2. Materials and methods

### 2.1. MRH histology and LSM

All animal procedures were conducted under guidelines approved by the Duke Institutional Animal Care and Use Committee. Specimens were perfusion fixed using an active staining method that has been described in detail previously (Johnson et al., 2019). Warm saline to flush out blood was perfused through a catheter in the left ventricle. This was followed by a formalin/Prohance (Gadoteridol) mixture titrated to reduce the spin lattice relaxation time (T1) of the tissue enabling accelerated scanning. The MRH scanning was performed on a 9.4T vertical bore magnet with a Resonance Research Inc. (Billerica, Md) gradient coil yielding peak gradients up to 2,500 mT/m. The scanner is controlled by an Agilent console running VnmrJ 4.0. The acquisition was accelerated using compressed sensing (Wang et al., 2018b; Johnson et al., 2019). Diffusion tensor images were acquired using a protocol described in detail in Johnson et al. (2022). The protocol employed a Stesjkal Tanner spin echo sequence with *b*-values of 3,000 s/mm^2^, 108 angular samples spaced uniformly on the unit sphere, a compression factor of 8 × yielding a large (252 GB) 4D volume with isotropic resolution of 15 μm. A baseline (b_0_) image was acquired after every 10th angular sample, yielding 18 baseline volumes. These volumes were averaged together to create a template to which all other volumes were registered (ANTs) to correct for residual eddy currents. A MATLAB script produced a diffusion weighted image (DWI) by averaging the 108 diffusion images together. The 4D data volume was processed through DSI Studio^1^ using both the DTI and GQI algorithms (Yeh et al., 2010) which yields eleven different scalar images (see Supplementary Table 3). We explored the use of the following DTI scalar images to drive the registration: axial diffusivity (AD), diffusion weighted (DWI), fractional anisotropy (FA) and radial diffusivity (RD). Two scalar data sets (DWI and FA) were used to registered labels to the MRH volumes (and thence to the LSM) using the Small Animal Multivariate Brain Analysis (SAMBA) an pipeline described fully in Anderson et al. (2019).

Five specimens from Johnson et al. (2022) were included in this study. They are summarized in Table 1. Specimen 200316, a 90 day male C57/B6 mouse was used as a reference atlas. It provides a modified version of the Common Coordinate Frame (CCFv3) from the Allen Brain Atlas (Wang et al., 2020). The CCFv3 defines regions of interest (ROIs) for 461 structures. Many of these structures are so small that reliable alignment is challenging. The reduced CCFv3 (rCCFv3) is a set of 180 labels/hemisphere generated by combining some of the regions in CCFv3 that are too small to transfer accurately in the registration pipeline. The full summary of the rCCFv3 can be found in Johnson et al. (2022).

**TABLE 1:** Test specimens for combined magnetic resonance histology (MRH)/light sheet microscopy (LSM) registration.

Specimen	Strain/Age	Fiducial	NeuN	Syto	MBP	IBA1	AutoF
191209	C57/90 d	175	X	X	X		
200302	C57/90 d	50	X		X		X
200316	C57/90 d	200	X		X		X
190108	BXD89/111 d	52	X	X		X	
200803	BXD89/687 d	51	X	X		X	

Following the MRH scans, the brains were removed from the skulls and sent to LifeCanvas Technology^2^ for tissue clearing and LSM imaging. The brains were cleared using SHIELD (Park et al., 2019) and stained using SWITCH (Murray et al., 2015) and scanned on a selective plane illumination microscope (SPIM) yielding three channel whole brain images at a resolution of 1.8 × 1.8 × 4.0 μm. Each of the three channels yields a nearly isotropic volume at a different wavelength of ∼ 300GB. The aggregate dataset for one specimen (MRH and 3 channels of LSM) is typically ∼ 1 TB. Table 1 lists immuno histochemistry stains that were used to test the pipeline.

### 2.2. Multiple stages of the workflow

**FIGURE 2:**
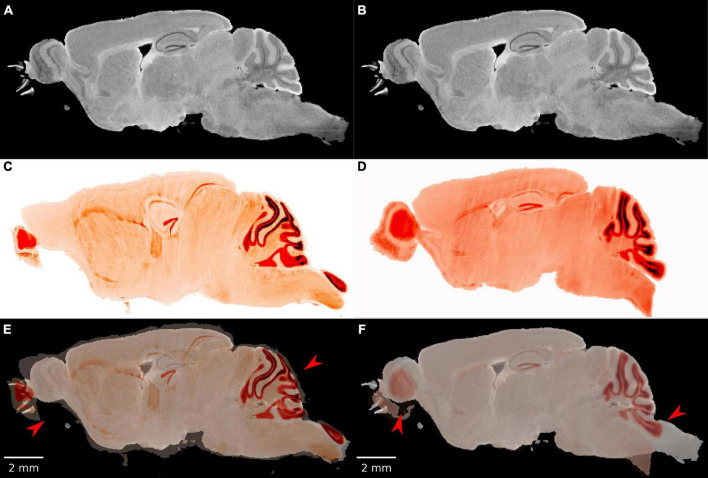
The failure of existing registration algorithms in the cerebellum and olfactory bulb. **(A,B)** DWI; **(C,D)** NeuN image after registration; **(E,F)** overlaid DWI/NeuN (specimen 191209). The left hand column shows the result of Elastix (Klein et al., 2010) with rigid and b-spline registration and default settings. The registration errors in the olfactory bulb and brain stem are reduced but the errors in the dentate gyrus and cerebellum are significant (arrows in panel **E**). The right hand column shows the result of ANTs (Avants et al., 2008) with affine and SyN and default settings. There is a reasonable overlap in the dentate gyrus but significant mismatch in the cerebellum and olfactory bulb (arrows in panel **F**).

Initial attempts at registration with popular registration algorithms (Avants et al., 2008; Klein et al., 2010) were particularly unsuccessful in cerebellum and olfactory bulb both of which are prone to significant distortion after removal from the skull (Figure 2). Our workflow employs an initial manual initialization followed by an automated multistep registration based on ANTs (Avants et al., 2008). The manual initialization is applied to all specimens to correct the most challenging distortions. It uses sparse landmarks (15∼20) with many concentrated in olfactory bulb and brain stem where the tissue distortion in the LSM are the greatest. Landmarks are placed in pairs, on both LSM and MRH. The landmark locations are 4 landmarks on olfactory bulbs, 2–3 landmarks on vessels on both sides between cortex and striatum, 3 landmarks on cerebellum, 2 landmarks on dentate gyrus, 2 landmarks on hippocampus and 2 landmarks on brain stem (as shown in Supplementary Figures 5B, D). The second automated step is described in detail below.

### 2.3. Quantitative loss function

The goal of registration is to transform the image of interest, M i.e., the image that is being moved (the LSM volume) into the space defined by the fixed reference image F (MRH volume). Our pipelines use a series of transforms applied successively with a loss function to evaluate each stage of transformation. For a single transform stage n, the transformation T_*n*_ can be obtained from optimizing the loss function:


(1) $$L_{n}(M,F)=S(T_{n}\circ M,F)$$

in which S is the similarity between F and transformed M. Common similarity metrics include mutual information (MI), cross correlation (CC), mean square error (MSE), which capture how well the two images are matched based on the joint histogram or signal intensities. Since we may use these metrics during registration, using the same metric repetitively for evaluation is unacceptable. At the same time, MSE, CC, global MI etc., by their intensity-based or histogram-based principles will not generate a stable predictability map between LSM and MRH due to the wildly different contrasts. The further explanation can be seen with the MI equation in the section “2.4 Optimization and validation.” Therefore, we need to devise a different loss function.

The initialized LSM data is warped to MRH space with a combination of registration steps built on ANTs (Avants et al., 2008). Our workflow encompasses multiple types of registration, and each type has different settings of metrics for optimization and multi-resolution coarse-to-fine refinement. The loss function should evaluate the cumulative consequences of each of these steps. We devised a loss function based on a large group (50–200) of fiducials to optimize the pipeline and evaluate its stability (see Table 1). We emphasize that these fiducials were used only in the evaluation of our pipeliness and are not required for routine use. These fiducials were generated by an experienced researcher on five different specimens (see Table 1) and consisted of matched pairs of points in MRH and LSM. Assuming the composite transform generated from our workflow is T, applying T to the fiducials in the space of LSM transforms these fiducials to the MRH space. The distance between one MRH fiducial (r_mr_) and its corresponding transformed LSM fiducial (T(r_lst_)) in the space of MRH is regarded as displacement from ground truth, and the average displacement i.e., L2 norm is used as the loss score, i.e.


(2) $$\text{L}2=\frac{{\sum_{\text{i}=1}^{\text{n}}\left({\text{r}}_{\text{mr,i}}-\text{T}\left({\text{r}}_{\text{lst,i}}\right)\right)}^{2}}{n}$$

### 2.4. Optimization and validation

The registration transform can be separated into linear and non-linear stages. To reduce the computation, a complicated registration should start from the linear transforms to adjust the position, orientation, and scaling of the moving image to coarsely and globally match the fixed and moving images. Then, application of non-linear transforms will deform the grid to locally match the fine details of fixed and moving images. From the popular options of non-linear transforms, we choose b-spline and symmetric diffeomorphic normalization (SyN) registration methods based on their efficiency on large datasets with complicated geometry.

B-spline relies on the control points to adjust local transform until reaching the minima of the loss function. The curve defined by b-spline is a conjunction of multiple polynomial curves which only depends on a local group of the control points. Based on the zero-order parametric continuity of B-spline, changing one control point will only influence the local neighborhood on the grid instead of propagating further. Therefore, b-spline can generate localized deformations flexibly and is computationally efficient when dealing with many control points. The conventional b-spline method applies free-form deformation to the image. In this study, the reversal form of the deformation is also required when transforming images between the fixed and moving spaces. Hence, we adopt the b-spline with the explicit symmetry i.e., b-spline Syn (Tustison and Avants, 2013) in the actual practice.

SyN, as a representation of diffeomorphic algorithms, generates voxel-wise transformation based on symmetrical and invertible displacements and velocity fields. SyN is implemented on the Insight ToolKit platform and based on Large Deformation Diffeomorphic Metric Matching (LDDMM) principles. As an improvement, it develops the symmetry between the fixed and moving images, i.e., instead of maximizing the similarity between *T*°*M* and F, SyN maximizes the similarity between φ_1_(*m*,*t*)*M* and φ_2_(*f*,1−*t*)*F*, in which *t* ∈ [0,1], m and f are the respective identity positions of M and F, and φ_1_, φ_2_ are the respective correspondence maps from M to F, and from F to M. Based on the backward and forward symmetry, *t* = 0.5. The optimization problem is then based on the equation:


(3) $$\begin{array}{l} E(F,M)\\ =\underset{\varphi_{1}}{\inf}\,\underset{\varphi_{2}}{\inf}\int_{t=0}^{0.5}\left\{{\left\|v_{1}(x,t)\right\|}_{L}^{2}+{\left\|v_{2}(x,t)\right\|}_{L}^{2}\right\}dt\\ +S_{\Omega }\left(\left|F\left(\varphi_{1}\left(0.5\right)\right)-M\left(\varphi_{2}\left(0.5\right)\right)\right|\right) \end{array}$$

to minimize both the pixel displacement and the difference between *F*(φ_1_(0.5)) and *M*(φ_2_(0.5)), in which υ_1_ and υ_2_ are velocity fields in the opposite directions, *S*_Ω_ is the similarity measurements across the whole *x* surface. The advantage of SyN is the low computational cost and the preservation of the image topology.

An additional factor influencing the registration is the selection of the similarity metrics. The most common similarity metrics include cross correlation (CC) and mutual information (MI).

A common definition of CC is


(4) $$CC(F,M)=\frac{\sum_{i,j}\left(F_{i,j}-\bar{F})(M_{i,j}-\bar{M}\right)}{\sqrt{\sum_{i,j}{\left(F_{i,j}-\bar{F}\right)}^{2}}\sqrt{\sum_{i,j}{\left(M_{i,j}-\bar{M}\right)}^{2}}}$$

CC is very sensitive to significant rotation and scale changes and any intensity difference, which limits its performance on cross modality registration evaluation, but including local neighborhood CC into the optimization penalty may still help with matching the contours of cross modality images.

MI defined by:


(5) $$\begin{array}{l} MI(F,M)=H(M)-H(M|F)=H(M)+H(F)\\ -H(FM)=\sum_{m\in M}\sum_{f\in F}p(f,m)log\frac{p(f,m)}{p(f\,)p(m)} \end{array}$$

originates from information theory and measures how much information of one image can be predicted correctly from another image which is already known. In this equation, H is the entropy, p(f, m) is the joint probability density function of the fixed reference atlas F and the moving image M that is being mapped into that reference, and p(f) and p(m) are the marginal probability density functions of F and M.

MI is commonly used for cross-modality registration because it is based on intensity probability distribution instead of pure intensity. However, for registering MRH and LSM, only employing MI may be risky. As shown in Figure 1, e.g., DWI and NeuN, in regions like cerebellum and olfactory bulbs, the intensity of gray matter in DWI is relatively low while in NeuN is high; meantime, in the central parts of the brain and the cortex, the intensity in DWI is relatively high while in NeuN is low. With the definition of MI, the joint histogram of F and M is scattered and the MI in this case is low, with the minimum being 0 which means no mutual information between two images. MI is a good measurement for Image F,M when the joint histogram of F and M consists of one or multiple condensed distributions, but may not be a good similarity measurement for MRH+LSM as the local contrast distribution is wildly different. Therefore, if the loss function calculated by MI is high, we do not know whether it is induced by the geometric mismatch because of the failed registration, or just the local contrast difference between MRH and LSM.

Table 2 describes the steps for optimizing the registration between an MRH and LSM. In our initial tests we used the DWI and Syto16 images from specimen 191209, because they both present abundant landmarks with some similarities, though the contrasts are different. In later studies, we used DWI and NeuN because NeuN and Syto16 have similar contrast and the NeuN stain from LifeCanvas was more consistent. Table 2 lists multiple stages starting with the global alignment progressing to local higher resolution details. At each stage multiple variations of the ANTs modules appropriate for that task are compared. We refer to a collection as a “pipe” e.g., P1_01 is one combination of ANTs modules to perform global registration. The pipe with the lowest L2 norm is chosen for the final pipeline. The output of this pipe is the starting point for the next stage. The Syto LSM image was initialized using the coarse (20 point) landmark initialization correcting the large distortions in brainstem and olfactory bulb. The optimization described in Table 2 was performed on data that had been down sampled to 45 μm to allow a broad search of parameters. In each stage, we employ the multi-resolution method, which initially performs the registration at a lower resolution with fewer control points and then samples the control points to a higher resolution following convergence of the loss function without consuming large computing resources.

**TABLE 2:** Pipeline optimization pyramid @ 45 μm resolution.

Experiments	Optimization composition	Score
Stage 1 Global	To optimize the combination of multiple transforms	
P1_01	Affine (Default) + Syn (Default)	0.3467
P1_02	Affine (Default) + B-spline Syn (Default) + Syn (Default)	0.303
P1_03	Rigid (Default) + Affine (Default) + Syn (Default)	0.4269
P1_04	Rigid (Default) + Affine (Default) + B-spline Syn (Default) + Syn (Default)	0.3644
P1_05	Affine (Default) + B-spline Syn (Default)	0.3333
P1_06	B-spline Syn (Default) + Syn (Default)	0.3131
Stage 2 Similarity	To optimize the similarity metrics	
P2_01	Affine (MI) + B-spline Syn (CC) + Syn (MI)	0.303
P2_02	Affine (MI) + B-spline (CC) + Syn (CC)	0.3606
P2_03	Affine (MI) + B-spline (MI) + Syn (MI)	0.3385
P2_04	Affine (MI) + B-spline (MI) + Syn (CC)	0.3752
P2_05	Affine (CC) + B-spline (CC) + Syn (MI)	0.3186
Stage 3 B-spline	To tune the multiresolution setting in b-spline stage	
P3_11	- -shrink-factor 10- -smoothing 5	0.332
P3_12	- -shrink-factor 1- -smoothing 5	0.323
P3_13	- -shrink-factor 1- -smoothing 1	0.326
P3_21	- -shrink-factor 10 × 1- -smoothing 2 × 1	0.280
P3_22	- -shrink-factor 10 × 1- -smoothing 10 × 2	0.285
P3_23	- -shrink-factor 10 × 1- -smoothing 10 × 10	0.350
P3_24	- -shrink-factor 2 × 1- -smoothing 2 × 1	0.308
P3_31	- -shrink-factor 10 × 5 × 1- -smoothing 3 × 2 × 1	0.277
P3_32	- -shrink-factor 10 × 5 × 1- -smoothing 10 × 5 × 1	0.312
P3_33	- -shrink-factor 10 × 5 × 1- -smoothing 10 × 10 × 10	0.383
P3_34	- -shrink-factors 3 × 2 × 1- -smoothing 3 × 2 × 1	0.300
P3_41	- -shrink-factor 10 × 7 × 4 × 1- -smoothing 1 × 1 × 1 × 1	0.274
P3_42	- -shrink-factor 10 × 7 × 4 × 1- -smoothing 4 × 3 × 2 × 1	0.268
P3_43	- -shrink-factor 10 × 7 × 4 × 1- -smoothing 10 × 7 × 4 × 1	0.362
P3_44	- -shrink-factor 10 × 7 × 4 × 1- -smoothing 10 × 10 × 10 × 10	0.495
P3_45	- -shrink-factor 4 × 3 × 2 × 1- -smoothing 4 × 3 × 2 × 1	0.278
P3_51	- -shrink-factor 9 × 7 × 5 × 3 × 1- -smoothing 9 × 7 × 5 × 3 × 1	0.292
P3_52	- -shrink-factor 9 × 7 × 5 × 3 × 1- -smoothing 5 × 4 × 3 × 2 × 1	0.355
Stage 4 B-spline distance	To tune b-spline spline distance	
P4_00	Spline distance default to 26	0.268
P4_01	Spline distance = 10	0.341
P4_02	Spline distance = 40	0.268
P4_03	Spline distance = 60	0.268
Stage 5 Syn	Tuning the multiresolution setting in SyN stage	
P5_01	- -smoothing 3 × 2 × 1 × 0- -shrink 4 × 3 × 2 × 1	0.2679
P5_02	- -smoothing 10 × 7 × 4 × 1- -shrink 10 × 7 × 4 × 1	0.3543
P5_03	- -smoothing 10 × 7 × 4 × 1- -shrink 4 × 3 × 2 × 1	0.3084
P5_04	- -smoothing 1 × 1 × 1 × 1- -shrink 4 × 3 × 2 × 1	0.2703
P5_05	- -smoothing 0 × 0 × 0 × 0- -shrink 4 × 3 × 2 × 1	0.2637
P5_06	- -smoothing 0 × 0 × 0 × 0- -shrink 6 × 4 × 2 × 1	0.2626
P5_07	- -smoothing 0 × 0 × 0 × 0- -shrink 10 × 7 × 4 × 1	0.2625
P5_08	- -smoothing 0 × 0 × 0 × 0 × 0- -shrink 20 × 15 × 10 × 5 × 1	0.2633
P5_09	- -smoothing 3 × 2 × 1 × 0- -shrink 10 × 7 × 4 × 1	0.2656

Specimen is 191209-1-1. The steps and parameters for the pipes that were tested are summarized for each stage. For each stage only the parameters to be optimized will change, and one optimal pipe will be selected among the pipes within one stage. The aim of the pipeline initialization is to select an optimal registration variables for certain contrasts in MRH/LSM. The pipeline optimization has been performed using one specimen. The application to additional specimens and contrast combinations has been demonstrated in Supplementary Table 2.

The optimization pyramid (Table 2) includes:

∘ Stage 1 focuses on optimizing large *global* details. Each pipe employs linear registration (rigid and affine) followed by non-linear registration (b-spline syn and syn). Each pipe uses the same default parameters. In stage 1, P1_02 i.e., Affine (Default) + B-spline Syn (Default) + Syn (Default) yielded the lowest loss score so its output served as the input for stage 2.
∘ Stage 2 focuses on *similarity metrics*, i.e., mutual information or cross correlation.
∘ Stage 3 adjusts the *b-spline multi-resolution* settings with number of layers, shrink factors (i.e., down-sampling) and smoothing sigmas (i.e., the radius of Gaussian filter).
∘ Stage 4 adjusts the *b-spline distance*, an additional parameter in b-spline syn.
∘ Stage 5 alters the *syn multi-resolution* settings with different number of layers, shrink factors and smoothing sigmas.

The pipe with the lowest L2 norm is labeled in green at each stage.

### 2.5. Registration validation

Registration with the five specimens was evaluated using the fiducials recorded in Table 1. The use of fiducials facilitates the comparison of different pipes and image combinations explained in the section “3.1 Optimization of pipes” and “3.2 Pipeline performance with varied image combinations.” Supplementary Figure 5 shows the dense collection of fiducials used to optimize the pipes (specimen: 191209). We performed an initial evaluation on specimen 200316 with an equally dense set of fiducials. At this point, it was clear that a sparser set would be adequate for validation in the other specimens.

The precision of a given registration was measured using Imaris^3^ which allows one to load multiple 3D volumes of different spatial resolution as layers. Vascular landmarks were identified using the three-plane view. Imaris allows one to toggle between an LSM image and a companion MRH image while interactively moving a 3D cross hair. One initially identifies a vessel in cross section in the LSM and moves the plane until one encounters a bifurcation. At this point the 3-dimensional coordinates are recorded. The process is repeated in the MRH and the Euclidean distance is measured. Supplementary Figure 3 shows the magnified cross section of a vessel in the NeuN image. The plane of the vessel cross section was adjusted until the bifurcation was evident and a fiducial was marked. The RD image provides high contrast for the same vessel where the same vessel bifurcation is visible.

### 2.6. Data and code availability

We have made the data for experiments 1--3 available under creative commons by NC-SA at https://civmimagespace.civm.duhs.duke.edu/login.php/client/4. The data is stored in H5 format to enable interactive examination using Neuroglancer.^4^ Reviewers can log in with the following credentials. Viewers will remain anonymous. cr371@duke.edu

Password: mrmicroscopy

The code is available in github.^5^ The code provided is implemented in Perl and bash (which are available on windows/macos) and based on Ants.^6^

When applying this method, please follow the procedures described in the accompanying instructions for installation and in the method section. The processing time will depend on the computing resource. Please use a high-performance computing resource paired with high memory and page faulting, especially if the input data is hundreds of GB.

## 3. Results

### 3.1. Optimization of pipes

**FIGURE 3:**
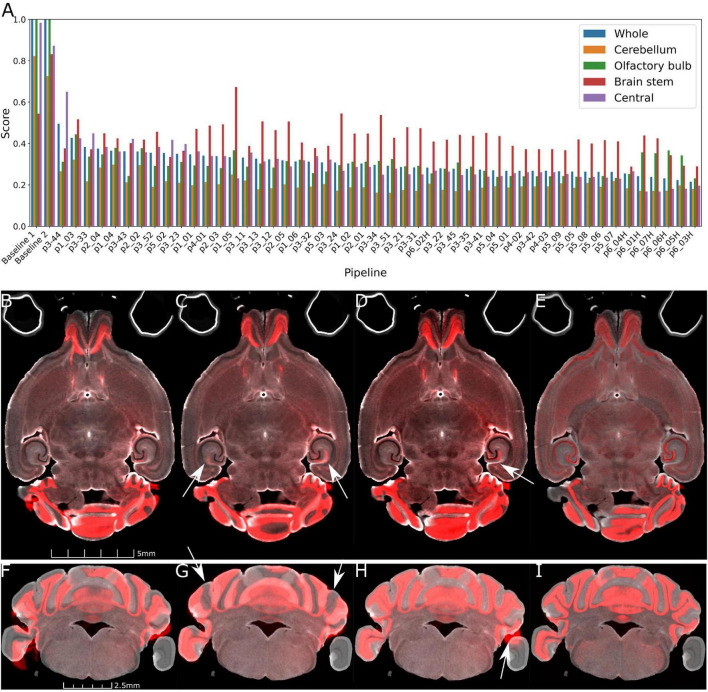
Demonstration of the range of results derived from the varied pipes. **(A)** Shows the L2 norm for the pipes listed in Tables 2, 3 registering Syto16 to DWI (specimen 191209). **(B)** Shows that the initialization results in reasonable alignment in the central slice. But **(F)** shows that initialization fails in the distal slices in the cerebellum. **(C,D,G,H)** Show results at 45 μm with L2 norms of 0.361 using pipe P2_02. There are still significant errors in the cerebellum (arrows in panel **G**). **(D,H)** With pipe P3_42 performs better with a lower L2 norm of 0.268. Finally, a comparison of panels **(D,H)** (@ 45 μm) and **(E,I)** (@ 15 μm) with pipeline P6_07_H demonstrates the utility of performing the registration using the higher resolution data. The cerebellar slice in panels **(F–I)** highlights a frequent problem i.e., loss of the parafloculoss from handling. The broken symmetry in the data gives rise to asymmetric misalignment (arrows in panels **C,D,H**).

Figure 3A plots the rank ordered L2 norm for each pipe. Visual comparison are provided in Figures 3B–I. Figures 3B, F, the starting point for all the comparisons shows the initialization using ∼20 manual landmarks. The comparison between a 45 μm pipe that is less accurate (e.g., p2_02, L2 = 0.361) and the optimal pipe @ 45 μm e.g., (p3_42, 0.268), is shown in Figures 3C, D, G, H. The improvement is evident (see white arrows in Figure 3G).

The parameters derived from Stages 1–3 had significant impact on the L2 norm. Changing the b spline distance and Syn in Stages 4 and 5 had less impact so the default settings were used in P3_042 as the starting point for experiments conducted with the full resolution (15 μm data) outlined in Table 3. The variable of interest for this stage of optimization is the shrink factor. This last stage is more nuanced depending on compute time and the combination of LSM/MRH contrasts (e.g., DWI/Syto, FA/NeuN) which is discussed in more detail in the section “3.2 Pipeline performance with varied image combinations.” The optimization @ 15 μm is started from pipe P6_01, which has the same registration setting with the optimal pipe @ 45 μm (P3_042). Table 3 demonstrates that the shrink factor has an enormous impact on compute time but the L2 norm remains relatively unchanged. Inspection of the results shows more subtle impact of the shrink factor. P6_01H overfits the data and is 27 times slower. P6_07H does not overfit and it can be executed in a modest time. Comparison between the best pipe at 45 μm (P3_042) and P6_07H optimized on 15 μm is shown in Figures 3D, E, H, I.

**TABLE 3:** Optimization of pipeline @ 15 μm resolution.

Pipeline	Composition	Score	Time
**Stage 6**			
P6_01_H	- -shrink-factor 10 × 7 × 4 × 1	0.2419	3 d 17 h
P6_02_H	Coarser affine - -shrink-factor 30 × 21 × 12 × 1	0.2834	6 d 12 h
P6_03_H	- -shrink-factor 30 × 21 × 12 × 3	0.2147	2 h 29 m
P6_04_H	- -shrink-factor 30 × 21 × 12 × 1	0.2544	6 d 12 h
P6_05_H	- -shrink-factor 40 × 28 × 16 × 4	0.2232	2 h 43 min
P6_06_H	- -shrink-factor 20 × 14 × 8 × 2	0.2314	18 h 12 m
P6_07_H	- -shrink-factor 10 × 7 × 4 × 2	0.2382	10 h 29 m

The shrink factors in the b-spline and SyN stages are the main variables to be optimized.

The L2 norm is also shown separately for the cerebellum (CB), olfactory bulb (OB), central section of the brain (C), and brain stem (BS). Each region poses unique challenges to the algorithm. The contrast is very high between the white matter and the intensely stained granular cell layer in the cerebellum in both the NeuN and Syto images, and there is comparable strong contrast in the DWI. Thus, the L2 norm for this cerebellar region converges to a low value for all the pipes. In the central part of the brain, the dentate gyrus, fimbria, and corpus callosum all provide unambiguous landmarks and fine tuning the pipeline leads to gradual improvement in the score. The olfactory bulb shows a similar effect, but the score does not converge to as low *a*-value. This may be because the olfactory bulb is one of the most distorted regions of the brain, and there are frequent tissue tears (e.g., the top red arrow in Figure 1). Finally, the brain stem is the most challenging region for registration as evidenced by high L2 norm and the high variability between different pipes. The cause of this is again evident on inspection of the sagittal LSM and MRH imaged in Figures 1C, 2D, F. The spinal cord in the LSM is grossly misplaced from its natural position forcing the algorithm into large displacements.

The transform obtained from the 15 μm registration was applied to the full resolution LSM data through the python interface of 3D Slicer, in the order of their generation. The time to apply transforms to full resolution LSM data (∼300GB) was ∼2 h.

### 3.2. Pipeline performance with varied image combinations

**FIGURE 4:**
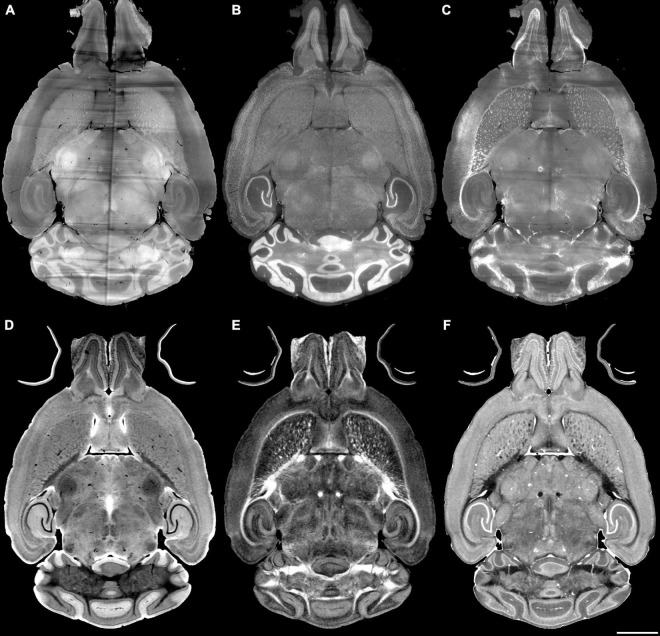
Light sheet microscopy of different stains and MRH of different contrasts. **(A)** Auto fluorescent, **(B)** NeuN, **(C)** MBP, **(D)** DWI, **(E)** FA, **(F)** RD scale bar is 2 mm (specimen 200316).

The registration success depends on the similarity between the anatomical features that are evident in the fixed and moving volumes. The initial work described above varied the pipes while registering Syto16 to DWI using specimen 191209-1-1. This section of the manuscript uses a fixed pipe (p6_07H) to explore the success of several specific combinations of LSM/MRH images in another specimen (200316) to demonstrate the approach more broadly. The DTI pipeline produces eleven different scalar images, each highlighting different diffusion properties (see Supplementary Table 3). The anatomic landmarks in the LSM vary widely depending on the immunohistochemistry used. There are an enormous number of combinations. Figures 4A–F show representative comparisons derived from specimen 200316 to help justify the comparisons we chose. The auto fluorescence (AutoF) image (Figure 4A) is frequently used to drive registration to the AutoF image in the ABA. NeuN (Figure 4B) and Myelin basis protein (i.e., MBP, Figure 4C) are of particular interest to our work in aging. The DWI (Figure 4D) is created by averaging all the (registered) diffusion weighted images producing high contrast to noise with many anatomic landmarks throughout the volume. Cortical layer definition and contrast in the dentate gyrus are particularly high in this volume. There are strong similarities between NeuN (Figure 4B) and DWI (Figure 4D). The FA image (Figure 4E) is a logical choice as it highlights white matter. The RD image (Figure 4F) is a putative marker of myelin integrity that might map well to the MBP.

#### 3.2.1 Comparison of p6_03H and p6_07H

Two pipes were chosen for more careful comparison: p6_03H and p6_07H. Because of the similarities between NeuN and DWI, this combination was chosen to evaluate these two pipes in three different specimens. Supplementary Figure 1 and Supplementary Table 1 summarize the comparison. P6_03H is faster than p6_07H and for one specimen (191209) yielded a lower L2 norm. The resulting volumes were imported into Imaris to allow interactive review of the relative success of the registration across the entire volume. Supplementary Figure 1 demonstrates that p6_03H yields consistent subtle misregistration in the dentate gyrus that is absent in p6_07H.

#### 3.2.2 Relative success of multiple combinations

Supplementary Table 2 summarizes an exhaustive comparison of p6_07H across five specimens with 15 different pairs of images. Specimen 200316 with the largest number (200) of fiducials was run twice with different initializations. Specimens 190108 and 191209 are from the BXD series providing a strain with different anatomy than the B6. Comparison of the L2 norms between specimens is not appropriate since each specimen has a different set of fiducials. This highlights some of the limitations in using fiducials as a quantitative metric for comparison of the quality of a registration. The precision of fiducial pairs will be biased by the reader placing the pairs. This results in a lower (nonzero) level which will vary between specimens that is dependent on the reader/fiducial e.g., an average error of 135 μm for the NeuN/DWI combination for specimen 200803 with 51 fiducials and 235 μm for specimen 191209 with 175 fiducials. However, comparison of the L2 norms across the different registration combinations within a specimen can provide useful insight into which pairs provide the best registration. For example, mapping MBP to RD is one of the least successful combinations. Mapping NeuN to DWI or Syto to DWI yields one of the lower L2 norms for all the specimens. The duplicate comparison for specimen 200316 highlights the stochastic nature of the registration with a 12% difference in the L2 norm (NeuN+DWI) between the two runs, but the relative scores of varied combinations of mapping remain unchanged.

One of the more surprising results is the success of the AutoF/DWI combination. Supplementary Figure 2 shows the results of registration using the pipe p6_07H with two image combinations: AutoF to DWI and NeuN to DWI with specimen 200316. The transforms generated with the AutoF to DWI registration was then applied to the NeuN. The registered pairs (NeuN to DWI) for both transforms were interactively reviewed in Imaris to discern areas in which the transforms differed. The target image (DWI) is displayed in yellow, and the moving image (NeuN) is displayed in green. In Supplementary Figure 2A (NeuN to DWI) there are subtle errors in alignment in the cerebellum that are not evident in the autoF/DWI pair. Yet the internal structures e.g., the dentate gyrus seem to be comparable. Comparison of the moving images C) NeuN or D) AutoF, highlight the high contrast granular layer in the NeuN image and the relatively flat contrast in the AutoF image. The high contrast in this granular layer dominates the registration since the NeuN stain in the outer edge of the brain is nonexistent. Registration using the AutoF is more successful since the contrast in the cerebellum is quite flat. This highlights one of the most challenging aspects of this task i.e., the registration of two volumes with completely different sources of contrast.

The NeuN/DWI combination has become our standard method since many of our planned studies require insight into neuronal density. Landmark comparison of the vessels in the NeuN to DWI registration was undertaken using Imaris as described in the section “2.5 Registration validation” to gauge the quality of registration away from the edges. The process was executed on 11 different vessels spread throughout the brain. The mean displacement was 22 ± 14 μm.

### 3.3. Volume corrections to LSM

**FIGURE 5:**
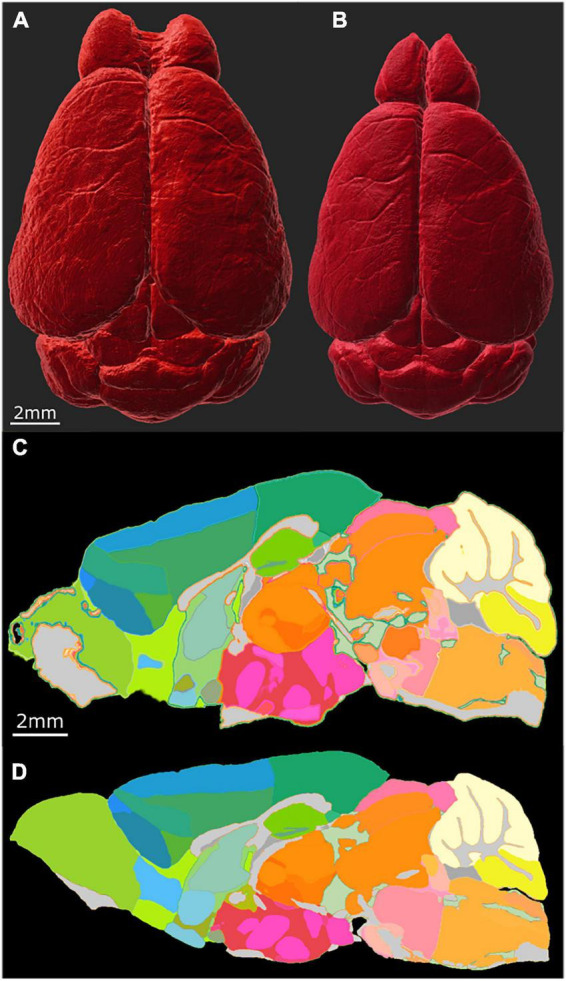
Distortion correction of the LSM data by registration to the MRI of the same specimen (190108). **(A)** Surface rendering of uncorrected LSM volume and **(B)** corrected LSM volume. The scale bar is 2 mm. **(C)** Midsagittal section of the labels on the LSM data before correction; **(D)** midsagittal section after correction: the scale bar in panels **(C,D)** is 2 mm. The distortion is present both within the plane of section and across the plane making it difficult to define identical planes. The highlighted edges in panel **(C)** are an interpolation artifact.

The most common way of delineating brain regions on an cleared brain image is *via* registration to an atlas (Kutten et al., 2016; Tappan et al., 2019; Perens et al., 2021) or registration of the atlas to the volume under study (Goubran et al., 2019). The most commonly used atlas is the ABA i.e., the CCFv3 3D template constructed from a population of 1,675 young adult B6 brains using AutoF (Wang et al., 2020). In Figure 5, we used our MRH atlas to estimate the regional volume changes in the LSM images from tissue swelling in specimen 190108. This specimen (111 day BXD 89) is representative of our broader interest- understanding the genetic basis for age related changes in the BXD family (Ashbrook et al., 2021). We registered the NeuN to DWI for specimen 190108 using the final registration pipeline. Labels were registered to the DWI of specimen 190108 from our reference B6 atlas (200302) using our MRH registration pipeline (Anderson et al., 2019). The transform that was generated was inverted to transform the labels on the DWI back to the uncorrected NeuN volume. Figures 5A, B shows the NeuN volume before and after correction, respectively. Note the changes in the width is larger than the change in length highlighting the nonuniform distortion. This is even more apparent in Figures 5C, D which shows a sagittal cross section before and after correction.

**FIGURE 6:**
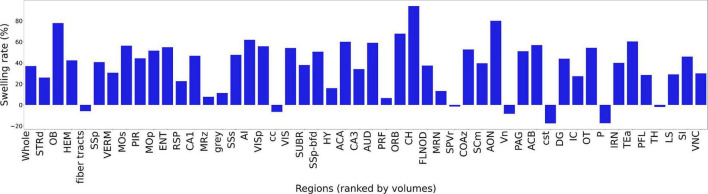
Bar plot of ratio of the volume before and after registration. The regions are ranked by the ROI volume. The ratio is obtained by $\frac{V_{b\,e\,f\,o\,r\,e\,r\,e\,g}-V_{a\,f\,t\,e\,r\,r\,e\,g}}{V_{M\,R}}$. The abbreviations of the regions are based on rCCFv3 (Johnson et al., 2022) with a labeling convention consistent with CCFv3.

Figure 6 summarizes the change in volume for the 50 largest regions of interest. We have used the reduced set of labels (rCCFv3) defined in Johnson et al. (2022). The nomenclature is consistent with CCFv3. The magnitude and variability are significant. The olfactory bulb (OB) is nearly 80% larger in the uncorrected data while the corpus callosum (cc) is ∼10% smaller. The problem is compounded when comparing specimens as the differential swelling varies, and it varies considerably between different clearing methods. These variations must impact the shape of the structures. Supplementary Figure 4 demonstrates the impact on the non-uniform distortion on the hippocampus, a region of particular interest in age related neurodegeneration (Sabuncu et al., 2011; Katabathula et al., 2021). Supplementary Figure 6 demonstrates the variability of deformation in 30 brain regions across multiple specimens.

## 4. Limitations

Registration of LSM to the MRH of the same specimen improves the geometric accuracy over existing methods of registration to the Allen Brain Atlas as demonstrated in Figure 6. But there are limitations. While the MRH data are acquired with the brain in the skull they are not a perfect match to the *in vivo* scan. Ma et al. (2005, 2008) have compared *in vivo* and *ex vivo* scans. They are significant with volume difference between *in vivo* and *ex vivo* (out of skull) varying from +60% (fimbria) to −79% (ventricles). The majority of this difference arises from removing the brain from the cranial vault. Our images have been acquired with the brain in the skull which reduces this problem. But the ventricles are collapsed and there may be shrinkage due to fixation. Inspection of the data before skull stripping has demonstrated no measurable separation of the brain surface from the skull so the shrinkage from fixation is limited. But ventricle distortion remains a limitation. An additional source of uncertainty arises from the transfer of the label from our canonical MRH atlas to any new MRH data using our SAMBA pipeline (Anderson et al., 2019). The accuracy and precision of the pipeline are dependent on the tuning parameters of the pipeline and the morphologic differences between the unknown specimen to which labels are mapped and the canonical atlas. We are confronted with the fact that the atlas is constructed from a B6 as is the ABA. But the tests performed in validating the atlas included a systematic variation of inputs using a synthetic model with varied anatomy and a real world source of variation based on a model of stroke causing significant volume changes in several structures in the brain. With appropriate selection of the SAMBA registration parameters ROC analysis showed area under the curve (AUC) better than 0.99.

## 5. Discussion

This work was initiated to enable combined analysis of cells and circuits from MRH and LSM in the same specimen. We have developed a method to register the LSM images which allow us to count cells to MRH, which maintains brain morphology inside the skull more closely approximating that in a live animal. Transferring labels from the MRH to the corrected LSM data allows us to measure regional cell densities with much greater accuracy than previous methods.

We addressed several challenges in correcting the significant and irregular distortion in the LSM; registration between fundamentally different images with significant differences in contrast; registration of very large volumes (300 GB). We have employed an initialization involving ∼ 20 landmarks followed by pipeline with multiple stages of transformations and metrics to minimize a user customized L2 norm score.

From the optimization, we selected the registration workflow with a combined consideration on accuracy and time. The optimized workflow (pipeline p6_07) takes an average of 7.5 h on a computer with 2 64-core processors and 2TB RAM with page faulting, with the L2 norm of 135 μm. The workflow shows robustness in multiple specimens. Our approach takes advantage of the high spatial and contrast resolution in the MRH images to provide internal landmarks the drive the registration locally across the whole brain which is evident from the small mean displacement (∼22 μm) of fiducials, which are picked at the junctures of vessels in both MRH and LSM.

As both MRH and LSM include varied contrasts (Figure 4), we did experiments to find the best combination of different diffusion scalar images and immunohistochemistry with LSM. A surprising conclusion is that registrations between DWI and AutoF or NeuN are similarly good. The practical consequence for our use is that we will not have to acquire an AutoF image freeing up a channel in the LSM for a more useful cytoarchitectural measure i.e., NeuN.

Multiple groups have developed methods for automated labeling of 3D optical images from cleared mouse brains (Kutten et al., 2016; Perens et al., 2021). These approaches rely on the Allen Brain Atlas as the reference (Wang et al., 2020). We are interested in mapping the age-related changes across multiple strains (for both genders). Registration of these data to the young adult male C57 that is the core of the ABA could obscure the morphologic changes of interest. Renier et al. (2016) used MRI of a fixed mouse brain to measure the degree of distortion from tissue processing with iDisco but their MRH images were of a half brain taken with a relatively low contrast gradient echo out of the skull. Labeling relied on mapping the autofluorescence image to the ABA. The MRI was not used in this step. Goubran et al. (2019) have developed a pipeline that is similar to that which we report here. Our work differs from their approach in four ways. Our dMRI protocols acquire data @ 15 μm vs 200 μm i.e., a difference in voxel volume of 2370 X with the commensurate challenge of larger image arrays. As demonstrated in Figures 3E, I, registration with the full resolution MRH (15 μm) makes a difference. Supplementary Table 2 provides an excellent starting point for evaluation of many of the alternatives. Finally, our pipeline takes advantage of a truly isotropic 3D MRH atlas of the brain in the skull to which rCCF3 labels have been mapped. Our approach provides an efficient method for segmenting brain regions in LSM data mapped in the MRH space of the same specimen which will allow quantitative study of cytoarchitecture e.g., cell density along with connectivity. The contrast study also would be a fruitful area for the further work. For example, a broader study could consider synthesizing synthetic contrast from combinations of scalar dMRI images that might contain complementary information or using machine learning to transferring the contrast from LSM to MRH to reduce the registration difficulty due to different contrast distributions (Sedghi et al., 2021). Artificial intelligence may well provide new avenues to improve the registration quality and efficiency (Fu et al., 2020; Sedghi et al., 2021).

## Data availability statement

The original contributions presented in this study are included in this article/Supplementary material, further inquiries can be directed to the corresponding author.

## Ethics statement

The animal study was reviewed and approved by the Duke Institutional Animal Care and Use Committee.

## Author contributions

YT, JC, and GJ contributed to the conception and design of the study. YT performed the investigation and implementation and wrote the original draft. JC and GJ organized the database. GJ reviewed and edited the submitted version and supervised and funded the study. All authors contributed to the manuscript revision and approved the submitted version.

## Footnotes

1
https://dsi-studio.labsolver.org/

2
https://lifecanvastech.com/

3
https://imaris.oxinst.com/products/imarisessentials

4
https://github.com/google/neuroglancer

5
https://github.com/YuqiTianCIVM/MRH_LSM_registration

6
https://github.com/ANTsX/ANTs
